# The Role of Maternal Smoking in Sudden Fetal and Infant Death Pathogenesis

**DOI:** 10.3389/fneur.2020.586068

**Published:** 2020-10-23

**Authors:** Nadja Bednarczuk, Anthony Milner, Anne Greenough

**Affiliations:** ^1^Department of Women and Children's Health, School of Life Course Sciences, Faculty of Life Sciences & Medicine, King's College London, London, United Kingdom; ^2^The Asthma UK Centre for Allergic Mechanisms of Asthma, King's College London, London, United Kingdom; ^3^National Institute for Health Research (NIHR) Biomedical Research Centre at Guy's & St Thomas' National Health Service (NHS) Foundation Trust and King's College London, London, United Kingdom

**Keywords:** sudden infant death syndrome - SIDS, sudden intrauterine unexplained death syndrome, hypoxia, hypercarbia, brainstem, carotid bodies

## Abstract

Maternal smoking is a risk factor for both sudden infant death syndrome (SIDS) and sudden intrauterine unexplained death syndrome (SIUDS). Both SIDS and SIUDS are more frequently observed in infants of smoking mothers. The global prevalence of smoking during pregnancy is 1.7% and up to 8.1% of women in Europe smoke during pregnancy and worldwide 250 million women smoke during pregnancy. Infants born to mothers who smoke have an abnormal response to hypoxia and hypercarbia and they also have reduced arousal responses. The harmful effects of tobacco smoke are mainly mediated by release of carbon monoxide and nicotine. Nicotine can enter the fetal circulation and affect multiple developing organs including the lungs, adrenal glands and the brain. Abnormalities in brainstem nuclei crucial to respiratory control, the cerebral cortex and the autonomic nervous system have been demonstrated. In addition, hypodevelopment of the intermediolateral nucleus in the spinal cord has been reported. It initiates episodic respiratory movements that facilitate lung development. Furthermore, abnormal maturation and transmitter levels in the carotid bodies have been described which would make infants more vulnerable to hypoxic challenges. Unfortunately, smoking cessation programs do not appear to have significantly reduced the number of pregnant women who smoke.

## Introduction

Sudden intrauterine unexplained death syndrome (SIUDS) is defined as the “sudden death of a fetus after the twenty-fifth week of gestation and sudden infant death syndrome (SIDS) the sudden death of an infant under the age of 1 year, which is unexplained following thorough examination of the clinical case, history, death scene and autopsy (1). Although the rates of SIDS have decreased during recent years due to successful public health campaigns (2, 3), the rates of SIUDS have remained largely unchanged and account for > 50% of stillbirths (1, 4–6). Despite modern advances, the pathophysiology remains unexplained, but is likely to be multi-factorial (7). The Triple Risk Model has been proposed as a potential explanation of SIDS; that is a vulnerable infant within a critical developmental period is exposed to an exogenous stressor (8). There have been many suggestions for the mechanism resulting in mortality including abnormal cardiorespiratory control, autonomic nervous system abnormalities and failure of arousal from sleep (7, 9, 10). Unfortunately, much less is understood about the pathogenesis underlying SIUDS, although there is likely to be an overlap (1).

Maternal smoking is a modifiable risk factor as both SIDS and SIUDS are more frequently observed in infants of smoking mothers (7, 11). A nationwide survey in New Zealand demonstrated smoking in pregnancy and/or bed sharing were the most important risk factors for sudden unexplained infant death regardless of ethnicity (12). A dose-dependent relationship exists, with an increasing risk of sudden death with increasing daily maternal cigarette consumption (13, 14). Between one and 20 cigarettes, the probability of sudden unexplained infant death increased linearly in one study. Furthermore, the risk was reduced in mothers who quit or reduced their smoking in pregnancy (14). If causality is assumed, 22% of sudden unexplained infant deaths in the United States of America can be attributed directly to maternal smoking in pregnancy (14). Worryingly, then the global prevalence of smoking during pregnancy is 1.7% with up to 8.1% of women smoking during pregnancy in Europe and 250 million women smoking during pregnancy worldwide (15, 16). Importantly, smoking cessation programs appear not to have significantly reduced the number of pregnant women who smoke (16–18). In a large randomised controlled trial involving 1,050 participants, nicotine patches did not increase the rate of abstinence from smoking until delivery or the risk of adverse pregnancy or birth outcomes, but compliance rates were low being 7.2% for nicotine patches and 2.8% for placebo patches used for more than 1 month (19).

The aim of this review was to highlight the role of maternal smoking in the pathogenesis of sudden fetal or infant death. We have, therefore, examined the literature to identify the mechanisms by which smoking in pregnancy could cause harm and the functional and structural abnormalities.

## Physiological Abnormalities in Infants of Mothers who Smoked

Newborns exhibit a biphasic response to hypoxia, with an increase in ventilation initially, followed by a later decrease in respiratory rate (20). In infants of mothers who smoked during pregnancy, a steeper ventilatory deceleration and a shorter time to reach the lowest oxygen saturations in response to hypoxia has been observed (20) and a greater decline in minute volume in response to a hypoxic challenge (21). These differences remained significant when adjusting for differences in birthweight, age and sex (20, 21). Furthermore, maternal smoking and substance abuse particularly impair the response to hypoxia in the prone position (22). This maladaptive response can be explained by the infant's chronic exposure to fetal hypoxia, leading to persistent, inappropriate activation of the respiratory neural network underlying the biphasic ventilatory response (21, 23). Furthermore, infants exposed to smoking *in utero* have been shown to display significant arousal abnormalities, taking longer times to wake when exposed to exogenous stressors, such as hypoxia, thus increasing vulnerability to SIDS (24, 25). Additionally, infants of smoking mothers also displayed a dampened ventilatory response to hypercapnia, which may be secondary to abnormalities within the central chemoreceptors, such as the locus coeruleus (26–28).

Abnormal cardiac function has been demonstrated in infants of smoking mothers. Increased vascular, cardiac and blood pressure reactivity in response to inhalation of 4% carbon dioxide or passive head tilt to 60 degrees has been demonstrated in infants of smoking mothers (29). The normal response to hypoxia is a tachycardia mediated by cardiac vagal motor neurons activated by lung stretch receptors (30). Infants of mothers who smoked during pregnancy showed a significantly lower increase in heart rate in comparison to infants of non-smoking mothers (20). Those findings could be explained by the enhanced inhibitory receptors on the smoke-exposed infant's heart and a dampened response to circulating catecholamines (31, 32). Nicotine exposure in pregnant rats led to a shift in expression of autonomic receptors in the offspring's heart, with a greater proportion of receptors with inhibitory effects on the heart (32). Consequently, the heart's response to circulating adrenaline was blunted by prenatal nicotine exposure (29, 32). In combination with an already reduced catecholamine production (31), the sympathetic response to stress following *in utero* smoke exposure was significantly dampened (33).

Perinatal exposure in mild 5-HT deficient rat neonates has been shown to exacerbate autoresuscitation failure (34).

## Mechanism of Harm Caused by Maternal Smoking

Tobacco smoke contains a variety of hazardous components, but its harmful effects are mainly mediated by the release of carbon monoxide and nicotine. Carbon monoxide can diffuse across the placental barrier and enter the fetal circulation. There it binds to fetal haemoglobin, causing an increase in carboxyhaemoglobin, resulting in a left shift of the oxygen dissociation curve and thereby reducing oxygen release into the fetal tissues (35). The resulting hypoxia in the fetus can be detrimental to development, especially for organs such as the lungs and brain (7, 36, 37). As a result of chronic hypoxia, aerobic cellular respiration is reduced leading to anaerobic metabolism and greater oxidative stress (38). This leads to higher levels of reactive oxygen species, which can directly damage and fragment DNA, ultimately resulting in apoptosis of developing cells (38, 39). Evidence of hypoxic injury in infants of smoking mothers is greater astrocyte gliosis (40, 41). Additionally, greater levels of iron accumulation in the central nervous system (CNS) have been shown; the iron may be the catabolic product of maternal methaemoglobin, a biomarker of oxidative stress (42). The combination of maternal anaemia and smoking appears to increase the risk of sudden perinatal death, likely by amplifying the hypoxic effect of smoking as maternal anaemia was not found to be an independent risk factor for sudden perinatal death (7, 43, 44).

Nicotine can enter the fetal circulation and affect multiple developing organs, including the lungs, adrenal glands and the brain (31, 45, 46). The damaging properties of nicotine are further enhanced by its prolonged half-life as a consequence of the immature fetal liver (33, 45, 47). Nicotine mimics the effects of the neurotransmitter acetylcholine (ACh) and acts on endogenous nicotinic ACh receptors (nAChR). Importantly, nicotine can cross the blood-brain barrier due to its lipid solubility and there have extensive effects due to the wide-spread expression of nAChRs in the CNS (18, 48, 49). In a review it was highlighted that the major brainstem sites where the expression level of nAChRs are consistently affected include those that play vital roles in cardiorespiration (hypoglossal nucleus, dorsal motor nucleus of the vagus nucleus of the solitary tract), chemosensitisation (nucleus of the solitary tract, arcuate nucleus) and arousal (rostral mesopontine sites such as the locus coeruleus and nucleus pontis oralis). Nicotine affects α7-nAChRs which play a vital role in the development of the brainstem regions receiving cholinergic projections in perinatal life (50). It can also bind to nACHRs in peripheral organs, such as the lung (45). In developing organs, the effect of exogenous nicotine on nAChR disrupts the natural sequence of cholinergic signalling, which is crucial for normal development, and can result in inhibition of DNA synthesis, abnormalities in gene expression and ultimately cell apoptosis (46, 51). This results in early termination of cell development and abnormal functioning of surviving cells (52). In the CNS, nicotine exposure has been shown to lead to neuronal hypoplasia, as well as an inappropriate early terminal differentiation of developing neurons, which is reflected in greater levels of expression of the post-mitotic cellular marker, neuronal nuclear antigen, in smoke-exposed victims (13, 53). Additionally, abnormal levels of neurotransmitters have been shown and abnormal neuronal function due to nicotine exposure (54, 55). These findings can be observed throughout the central nervous system, including the brainstem, cerebellar cortex, and spinal cord, but also in peripheral organ systems (45, 56). Hypoplasia of the pars compacta of the substantia nigra has been observed in SIDS victims; 77% of the SIDS victims had been exposed to maternal smoking; the substantia nigra pars compacta is involved in the sleep arousal phase (57).

### Central Nervous System (CNS)

#### Brainstem

Neuropathological studies of both SIUDS and SIDS cases have highlighted both hypoplasia, increased apoptotic figures and abnormal functioning in a number of brainstem nuclei (52, 56, 58). All these abnormalities have been found to be greater following smoking in pregnancy. Many of the affected nuclei are crucial in central respiratory control, for example the pre-Bötzinger complex in the ventral medulla and the pontine Kölliker-Fuse Nucleus (56). Both nuclei play an essential role in respiratory rhythm generation and in coordinating the change between inspiration and expiration (56, 59). The parafacial/facial complex is also required for respiration as it initiates the inspiratory phase of the respiratory cycle in conjunction with the pre-Bötzinger complex (60, 61). Hypoplasia of the parafacial/facial complex occurs predominantly in SIUDS cases, highlighting its importance in the progression to extra-uterine life (48, 56, 62). Further, the dentate-olivary complex and the dentato-rubro-olivary network, which drive the autonomic compensatory respiratory response to episodes of hypoxia, have also been implicated in sudden perinatal death (41, 63). Abnormalities in the arcuate nucleus of the hypothalamus, affecting cardiorespiratory responses and acting as a central chemoreceptor, have been found in > 50% of sudden perinatal death (64). A further vital structure affected in both SIDS and SIUDS is the raphe nucleus, a key producer of serotonin, which can modulate respiratory activity in response to changes in oxygen and carbon dioxide tension and pH levels (54, 65). It has also been proposed to influence intrauterine autonomic nervous system control with respect to vital homeostatic functions (66). Studies have shown a reduction in serotonin throughout the brainstem of sudden perinatal death cases. Nicotine significantly affects alpha 7-nicotinic acetylcholine receptors which have essential roles in the development of the brainstem regions receiving cholinergic projections in perinatal life. High alpha 7-nicotinin acetylcholine receptor expression levels were only observed in one study in the infants of mothers who smoked;, this was frequently associated with hypoplasia of brain structures involved in vital functions (46).

Brainstem nuclei mediating arousal such as the inferior colliculus and locus coeruleus have also been implicated in sudden perinatal death. The inferior colliculus plays an important role in processing auditory information, but also in the sleep-wake cycle (67). The locus coeruleus is the main source of noradrenaline in the brainstem and thereby coordinates multiple autonomic function such as the sleep-wake cycle and cardiorespiratory control (68, 69) and also acts as a central chemoreceptor for carbon dioxide (27, 70). Maternal smoking has been associated with greater rates of hypoplasia and abnormal neurotransmission in the locus coeruleus (68, 69). Specifically, reduced levels of noradrenaline and tyrosine hydroxylase, the enzyme necessary to produce noradrenaline, were observed (68). Nicotine exposure in a primate model produced brainstem and autonomic abnormalities of the key monoamine system that govern the response to hypoxia;. interestingly the effects were offset by coadministration of the anti-oxidant Vitamin C (71). A review has highlighted that the brainstem of infants who died from SIDS exhibited abnormalities in major neurotransmitter and receptor systems including catecholamines, neuropeptides, acetylcholinergic, serotonin, glutamate, brain derived neurotrophic growth factor and cytokines (72).

#### Cerebellar Cortex

The cerebellar cortex is particularly vulnerable to exogenous toxins due to its prolonged developmental period, commencing in early fetal life and continuing throughout the 1st year of postnatal life (73, 74). It plays not only a key role in motor coordination, but also the coordination of cardiorespiratory and autonomic nervous systems, with multiple modulatory connections to the brainstem and the cerebral cortex. As such, cerebellar abnormalities can result in dysfunctional breathing following birth, enhancing the risk of sudden death (49). The two main neuronal cell types in the cerebellum, the Purkinje cells and the granule cells, show evidence of hypoplasia and immaturity in sudden perinatal death, with a significant correlation to smoke exposure. In a study of 21 cases of sudden fetal death and 25 cases of sudden infant death, a high percentage of developmental defects of the purkinje cells were noted (75). Further, abnormal trophic signalling has been evidenced with a significant reduction in Brain-derived neurotrophic factor (BDNF) in the cerebellum of SIUDS/SIDS cases (76). These abnormalities in the developing cerebellar cortex found in SIUDS/SIDS cases were greater following maternal smoking in pregnancy (49).

A particular vulnerable area in the cerebellum to the effects of smoking is the external granule layer (EGL), which is present throughout fetal life, but following birth progressively thins and disappears (73, 74). This area expresses a significant number of nACHRs, highlighting the importance of cholinergic activity in the development and differentiation of EGL neurons. Exposure to tobacco smoke is associated with a significant reduction in the receptor level in sudden perinatal death cases (49).

#### Spinal Cord

Spinal cord abnormalities have also been implicated in the pathogenesis of sudden perinatal death and have been shown to be more frequent in infants of mothers who smoked during pregnancy (56). *In utero*, the spinal cord, in particular the intermediolateral nucleus, has been shown to initiate episodic respiratory movements that facilitate lung development (56). Greater rates of hypo-development of this nucleus have been observed in SIUDS and SIDS victims, particularly in infants of mothers who smoked during pregnancy (56, 77).

### Autonomic Nervous System (ANS)

The area postrema plays a key role in chemoreception and controlling autonomic functions. Neuronal hypoplasia, abnormal vasculature and reactive gliosis have been found in this area in both SIUDS and SIDS cases. Importantly, these abnormalities were more common in offspring of smoking mothers (78).

The carotid bodies can also be adversely affected by tobacco exposure. Smoke exposure can lead to abnormal maturation and neurotransmitter levels such as dopamine, leaving infants more vulnerable to hypoxic states (58, 79–81). These findings may further explain the impaired arousal observed in smoke-exposed infants in response to hypoxia (24, 25). A study has demonstrated a significant correlation between unexplained death, altered substance P staining and maternal smoking (82, 83). Another in study, serial sections of 84 brainstems from subjects ranging from 17 weeks gestation to 8 postnatal months of life demonstrated histological and immunohistochemical alterations in the choroid plexus of the fourth ventricle including hyperexpression of substance P and apoptosis (84).

## Effects on the Respiratory System

Pulmonary hypoplasia has been more frequently observed in animal models exposed to nicotine *in utero*, but not in humans (45, 85). This effect appears to be mediated by exogenous nicotine that leads to an over-expression of nACHRs in the pulmonary epithelium, which leads to abnormal cholinergic activity during lung development (45, 86). In humans, antenatal smoking has an adverse effect on airway development, small airways being affected more than large airways. This is important as such abnormalities are associated with an increased tendency to wheeze in early childhood and predisposes to an enhanced response to an enhanced response to viral infections in early childhood (85). Antenatal exposure to smoking, however, does not result in increased bronchial hyper-reactivity (85). Pulmonary function testing has not demonstrated that infant lung volume is affected by antenatal smoking exposure, other than due to the expected effects of smoking on somatic growth (85). Large epidemiological studies have demonstrated that antenatal smoking exposure increases wheezing in infancy (87) and in the first 2 years after birth (88). In school age children an increased risk of episodes of dyspnoea (89) and an increased risk of asthma and persistent wheezing up to the age of 15 years (90) have been reported. Increased numbers of nACHRs have been observed in fibroblasts in the airways and the pulmonary vasculature (45). In the airways, greater collagen deposition can lead to greater airway resistance. Additionally, increased fibroblast activation by exogenous nicotine in the vasculature can explain the greater rates of pulmonary hypertension and atherosclerosis in smoke-exposed infants (91–93). Respiratory neural network abnormalities, in particular of the arcuate nucleus, were frequently observed in SIUDS cases (86).

## Further Considerations

It remains difficult to predict which fetus or infant will be affected by sudden death. As an important risk factor, reduction in maternal smoking should remain a key focus in trying to reduce rates of sudden fetal or infant death. Anti-smoking laws, such as prohibiting smoking in public places, however, have not resulted in a significant decrease in sudden fetal or infant death (94). Furthermore, nicotine-replacement therapies do not significantly reduce maternal smoking rates (19). Importantly, nicotine replacement therapies still expose the developing fetus to nicotine and thus could be associated with an increase the risk of sudden perinatal death (15, 18). It is therefore, important to educate children regarding the harmful effects of smoking and which may prevent young women from starting smoking.

## Contribution to the Field Statement

Maternal smoking is a risk factor for sudden infant death syndrome (SIDS) and sudden intrauterine unexplained death syndrome (SIUDS). Disappointingly then the global prevalence of smoking during pregnancy is 1.7% and up to 8.1% of women in Europe smoke during pregnancy; 250 million women smoke during pregnancy worldwide. A literature review has been carried out to identify the mechanisms by which antenatal smoking may result in SIDS or SIUDS. This has demonstrated that infants born to mothers who smoke have an abnormal response to hypoxia and hypercarbia and they also have reduced arousal responses. The harmful effects of tobacco smoke are mainly mediated by release of carbon monoxide and nicotine which result in abnormalities in brainstem nuclei crucial to respiratory control, the cerebral cortex, and the autonomic nervous system. Furthermore, abnormal maturation and transmitter levels in the carotid bodies have been described which would make infants more vulnerable to hypoxic challenges. Sadly, smoking cessation programs appear not to have significantly reduced the number of pregnant women who smoke.

## Author Contributions

NB and AG: undertook independent literature reviews. NB: wrote the first draft. All authors were involved in critical review of the literature, production of the manuscript, and approved the final version of the manuscript.

## Conflict of Interest

The authors declare that the research was conducted in the absence of any commercial or financial relationships that could be construed as a potential conflict of interest.
